# Social Connection, (Im)material Gains and Experiences of Inclusion of Asylum Seekers’ and Refugees’ Volunteering in Glasgow

**DOI:** 10.1007/s12134-022-00979-6

**Published:** 2022-08-11

**Authors:** Niroshan Ramachandran, Zana Vathi

**Affiliations:** 1 School of Law, Criminology and Policing, Edge Hill University, St Helens Road, Ormskirk, L39 4QP Lancashire UK; 2 Department of Social Sciences, Edge Hill University, St Helens Road, Ormskirk, L39 4QP Lancashire UK

**Keywords:** Asylum Seekers, Refugees, Volunteering, Everyday Life, Materialities, Inclusion

## Abstract

Volunteering is an activity based on a non-profit idea of engagement in productive transactions. This paper examines why and how asylum seekers and refugees (ASRs) partake in volunteering focusing particularly on the everyday, mundane experiences of volunteering and the role of the material and financial gains as part of it. Data is drawn from 30 interviews conducted with ASRs from 15 countries residing in Glasgow, 20 interviews with the third sector and state agency staff, and supplemented by participant observation conducted in third sector organisations involved in ASRs’ integration and settlement. Despite the individual and situational differences, volunteering appeals to ASRs as it enables them to gain familiarity with and social connectivity in their new environs as well as supplements subsistence needs, providing material and financial benefits. These mundane and seemingly secondary gains from volunteering consist of the flesh of the otherwise abstract processes of inclusion, due to the symbolic and logistic significance they have in the ASRs’ lives.

## Introduction

Volunteering is “working, the putting in of time and energy, which one person does for another or for the public, out of free will, and with no material compensation similar in quantity or quality to market value” (Cohen, 2009, p. 523). The research in this paper focuses on two key points related to volunteering and asylum seekers and refugees (ASRs). Firstly, the mundane, everyday aspects of volunteering—the material and financial aspects. Secondly, the impacts of ASRs’ participation as volunteers on their inclusion and integration. We also discuss the importance of third sector organisations (TSOs) in providing volunteering opportunities for ASRs and how this benefits their integration and inclusion.

The topic of ASRs’ volunteering and its impacts on integration and inclusion has been underexamined in academic research. Nevertheless, while under-exploring the holistic benefits of volunteering, some have seen volunteering as a pathway to employment and as contributing to self-improvement (Yap et al., 2010; Vickers, 2016; Hassemer, 2020). Presently, academic literature focusing on ASRs’ experiences of volunteering in Glasgow is lacking. Understanding why ASRs volunteer and why they remain engaged in volunteering deserves exploration, particularly owing to the positive impact that volunteering is known to have on ASRs’ lives and wellbeing elsewhere in the UK and beyond (Wood et al., 2019; Hassemer 2020). The significant positive impact that volunteering has on ASRs’ lives and wellbeing is relevant considering the hardships they encounter upon migrating.

### The Context: the UK and Glasgow (Scotland)

In 2021, there were 48,540 asylum applications submitted in the UK (Home Office, 2022). This number is higher than the applications submitted during the so-called European Migration Crisis that happened between 2015 and 2016. At the end of 2016, the UK received 36,546 applications. Previously, the UK has seen the largest number of applications in 2002 when 84,132 applications were submitted. Although the current numbers of applications are approximately less than 50% of 2002, the current trend shows an increase in the number of applications submitted in the UK. Among the applications submitted in 2021, only 14,734 people were offered asylum and humanitarian protection in the UK, which is 30% of the total applications. In the meantime, there has been a backlog of asylum applications to be processed in the UK. At the end of 2021, 81,978 applications were awaiting an initial decision. This is worse than the applications awaiting an initial decision in 2018, which was 27,256. This reflects the UK’s reluctance in granting asylum to people who seek it.

In the UK’s political context, measures have been introduced in response to the rising numbers of asylum applications, and those restrictive measures have not necessarily reduced the number of asylum applications since the 1990s. Nevertheless, these restrictive policy measures have been undermining the quality of human life while also preventing integration. Zetter and Pearl (2000, p. 675) emphasised that these restrictive measures signify “a persecutory regime of welfare disentitlement and social exclusion”. Individuals in the asylum process have been facing enforced destitution due to restricted access to welfare benefits and prohibition from employment while the protracted asylum system contributes to uncertainties (Hynes, 2009).

The Immigration and Asylum Act 1999 paved the way for the no-choice compulsory dispersal of asylum seekers around the UK which geographically isolates and socially excludes asylum seekers from potential social networks (Hynes, 2009). Asylum seekers have been socially excluded and marginalised by “having no choice about which city and what type of accommodation to live in and then being relocated several times” (Hynes, 2009, p. 18). Also, the no-choice dispersal has been contributing to “a feeling of loss of control over their lives and a sense of liminality, or limbo, imposed by the process” (ibid). Furthermore, asylum seekers are excluded from employment, forced to depend on low levels of support and face destitution (Karamanidou & Folley, 2020). They are left with £5.84 per day or £40.85 weekly asylum allowance to manage their lives^1^ (UK Government, 2022a). The individual asylum seeker has been expected by the government to cover the cost of their food, clothing and sanitation needs within this meagre amount of support. This weekly allowance provided to an asylum seeker is approximately 50% of the UK government’s Universal Credit support for persons with low income which is equivalent to £84 (UK Government, 2022b).

Recently, the UK government introduced the Nationality and Borders Bill (2022)^2^ which will treat asylum seekers arriving in the UK without permission as criminals. Widely described as an anti-refugee bill, the legal or illegal route of an individual asylum seeker’s arrival will shape their asylum claim. Once it becomes an Act, it will reinforce the existing narratives around “deserving” or “undeserving”; in other words, a formal two-tier system that treats asylum seekers as “worthy” or “unworthy” will be introduced.

Moreover, gaining refugee status does not alleviate the challenges faced by asylum seekers in the UK. Refugees have been provided with temporary 5 years of protected status and they then must apply for settlement in the UK (Stewart & Mulvey, 2014). This has resulted in the in-direct earned settlement process that requires individual refugees to prove that they require the continued need for protection in the UK and show good character. In addition, the refugees are facing challenges in gaining qualified employment due to a lack of recognition of their higher education and professional qualifications and employment experiences gained in their countries of origin. Overall, policies and practices in the UK are reinforcing broader control and exclusion of ASRs in the UK (Phillimore & Goodson, 2006; Kidd, 2017). As a result, ASRs are facing increased levels of uncertainty about their future.

Glasgow (the area of focus in this article) is one of the largest dispersal areas in the UK and the only city in Scotland to accommodate asylum seekers (Mulvey, 2015; Strang et al., 2017). Since 2000, Glasgow has received approximately 10% of the total number of asylum seekers arriving in the UK (Scottish Government, 2018). There are nearly 5000 asylum seekers receiving asylum support in Glasgow (Convention of Scottish Local Authorities (COSLA), 2019). Although Glasgow is one of the cities in devolved Scotland, the Scottish government has no power over immigration matters and therefore must follow the UK government’s policies and practices. Importantly, while the Scottish government promotes integration from day one of ASRs’ arrival in Scotland, the UK government states that individuals can be involved in integration only once they have gained their refugee status, which is often protracted.

The UK’s hostile policies increasingly hinder ASRs’ integration and ability to rebuild their lives (Mulvey, 2015). Consequently, upon arrival in Glasgow, ASRs face difficulties such as having low or no income, housing problems, language barriers, discrimination in the labour market, a lack of social support networks and limited access to services. For instance, asylum seekers have been housed in socially deprived areas on the outskirts of Glasgow that are managed by private accommodation providers. Neither the Scottish government nor the Glasgow City Council could intervene in housing provision or rather have limited involvement. Furthermore, as previously mentioned, asylum seekers will not receive any monetary support from the Scottish government apart from the weekly asylum allowance provided by the UK Home Office. Thus, they depend on state and non-state support. This dependence has created a sense of isolation in which ASRs find themselves in a weak position and unable to act independently (Phillimore & Goodson, 2006; Mulvey, 2010).

In these circumstances, volunteering has been identified as a tool for enhancing migrants’ social integration and inclusion (Wood et al., 2019; Hassemer, 2020; Ramachandran, 2021). Considering the challenges of participating in mainstream society, ASRs have utilised volunteering as a coping strategy to help them feel part of the host society (Healey, 2006). Limited studies focusing on migrants’ volunteer involvement in the UK highlight that those who volunteer experience significant benefits in terms of confidence, development of skills, employment prospects, social inclusion and community cohesion (Hunt, 2008; Yap et al., 2010; Vickers, 2016).

While scholars have discussed ASRs achieving or improving some aspects of their lives (skills or social connections) through volunteering, little attention has been paid to the mundane features of volunteering, particularly the material and financial gains and how they shape ASRs’ everyday encounters in their host community. The material aspects of ASRs’ lives remain pertinent to their everyday existence and they value the acquisition of material objects, such as household items, furniture and cutleries (Ramachandran, 2021). As Noys (2016, p. 82) emphasises, there is a “wider ‘structure of feeling’” for material objects. Thus, we examine the relationship between volunteering and the associated benefits with a focus on the mundane, the materiality of volunteering, and links between volunteering and ASRs’ inclusion. To this end, we uncover the intimate and hidden-to-the-public eye aspects of volunteering.

### The Mundane and Material Aspects of ASRs’ Volunteering: Impact on Inclusion

Volunteering has been incorporated into ASRs’ everyday practices, where it has specific functions and meaning contributing to ASRs’ pursuits and aims (Ramachandran, 2021). Importantly, motivations for volunteering cannot be dichotomised between altruistic (Snyder & Omoto, 2008) or egoistic (Clary & Snyder, 1999) motives because motivations for volunteering are multifaceted (Haski-Leventhal, 2009; Wilson, 2012; Hassemer, 2020). For ASRs, volunteering activities can be routine, random, or complex, yet they link to migrants’ interaction in their society and form their everyday encounters. As Neal and Murji (2015, p. 813) emphasise, “the micro, the slight, the most mundane, and the banally ordinary practices, emotions, social relationships and interactions also reflect convergences with and manifestations of wider societal factors, forces, structures and divisions”. As we will argue, volunteering undeniably transforms ASRs’ everyday lives.

Volunteering further provides an opportunity for refugees to progress in life and affords them agency (Hunt, 2008). It can also counter negative perceptions of ASRs as lazy, and economic burdens, and can be a route to employment (Yap et al., 2010). Strang et al. (2016) indicated that volunteering creates pathways for positive relationships between locals and newcomers, while a recent study conducted on the subject of African refugees in Australia identified that volunteering positively influenced their wellbeing and contributed to a two-way process of integration (Wood et al., 2019). ASRs may engage in volunteering for opportunities and resources to build themselves a better future, achieve their moral and or legal citizenship status (Hassemer, 2020), and facilitate integration. However, the material and financial benefits of volunteering have not been considered in volunteerism research with a focus on migrants.

Although the focus of this paper is to examine ASRs’ mundane experiences of volunteering, it is important to highlight the role of TSOs. TSOs are local, small and volunteer-led organisations (Phillimore & Goodson, 2010), such as Maslows in Govan, Bridging the Gap in Gorbals, the Govan Community Project in Govan, Maryhill Integration Network in Maryhill, Scottish Refugee Council and so on. The extensive role of TSOs in assisting societal newcomers highlights the burden placed on them by the state, “where responsibility has fallen on voluntary and community organisations to fill gaps in statutory service provision” (Wren, 2007, p. 391). This is significant for ASRs, especially for asylum seekers, who have limited access to resources and support; most of them meet some of their needs only through TSOs (Mayblin & James, 2019). On the one hand, as claimed by Findlay et al., (2007, p. 57), TSOs could be seen as performing the role of a shadow-state as “these organisations often rely on state grants or contracts”. The UK government has been facilitating the delivery of support and services to ASRs by funding TSOs. Therefore, the TSOs could be seen as constituting a shadow-state apparatus by remaining within the arena of state regulation. This paper thus acknowledges the state’s response and its changing social-political dynamics in service provision considering the two-way process of volunteering.^3^ On the other hand, TSOs’ initiatives and practices to support ASRs move them away from purely performing shadow-state activities (ibid). TSOs share vital information with ASRs, provide advice/consultations, advocate for adequate state support, provide additional support to obtain services, and provide volunteering opportunities. Wren (2007, p. 409) emphasises that Glasgow’s responses to supporting vulnerable ASRs demonstrate “the advantages of coordinating and delivering services through the mechanism of multi-agency networks”. Mayblin and James (2019) further note that the UK government’s policy restrictions and lack of support are creating a gap in fulfilling ASRs’ needs that hinders their integration and settlement. Thus, where possible, TSOs attempt to fill gaps.

Migrants give significant value to materialness or thingness due to inadequate state support. Conlon (2011) finds that ASRs’ ability to accumulate new material possessions is at minimal levels due to the restrictions imposed through the asylum system. Wilson (2012) describes volunteering as a form of unpaid labour that depends on the consumable resources and the rewards. This is pertinent to this paper as attention is given to the relationship between volunteering and material and financial benefits. The use of materiality emphasises “the tangible attributes of the ‘material’” (Ho & Hatfield, 2011, p. 709) and materialities are integral to both personal and social aspects of migration. Dudley (2011, p. 247) notes that materiality “lies in the mutually constitutive relationships between people and things”.

ASRs’ everyday lives often unfold through material objects, which reveals the ambiguities brought by the immigration system (Conlon, 2011). Darling (2014) shows that mundane materials, such as letters, clothing or medications, have immense potential to materialise migration experiences significantly. He further highlighted that representations of ASRs are “translated in and through material things… through the relations they enact and the affects they produce” (Darling, 2014, p. 485). Furthermore, Dudley (2011) states that basic material available to refugees shapes their ability to develop new and creative ways to adjust to their new environment. Conlon (2011) further indicates that material objects that migrants acquire enable them to progress in their daily routines and connect with their new society. Thus, migrants’ ability to utilise basic material resources available to them in the process of the settlement provides a way to counteract passivity and dependency (Dudley, 2011; Ho & Hatfield, 2011).

The mundanity of ASRs’ volunteering also provides the platform for them to establish social capital to engage in collective resistance against the negative perceptions. It is because volunteering “is situated at, and builds bridges between, three levels: the community, the voluntary organization and the individual” (Hardill et al., 2007, p. 397), which this paper also reflects through various aspects of ASRs’ volunteering. Vickers (2016) emphasises that volunteering could lead to building oppositional social capital and developing wider collective identities. Although this paper is not focusing on collective resistance, Vickers’ findings on the positive impact of volunteering in reversing the negative perceptions and building social capital in the UK is significant.

This paper, through focusing on the mundane experiences of ASRs’ volunteering and material benefits, proposes that the material and financial benefits are important to ASRs’ volunteering motivations and so these benefits should be a crucial part to explore ASRs’ motivations to volunteer. Focal is how ASRs make sense of the material through volunteering and how material objects become the medium to express their motivations to volunteer and link to their broader strategies of survival, adaptation and inclusion.

## Method

The data for this article emerged from PhD research conducted in Glasgow between June and September 2018. During this period, 50 semi-structured interviews were conducted with asylum seekers, refugees and staff from TSOs and state agencies. Participants were recruited using purposive and snowball sampling methods, which are useful for identifying and selecting information-rich individuals or groups who have knowledge and experience suitable for the focus of a study (Patton, 2014), and when dealing with sensitive and hard-to-access populations, in this case, ASRs (Heckathorn, 2011; Dean et al., 2012). Different from the studies conducted on this topic, the sampling specifically focused on ASRs from different origins and at different stages of their asylum journey to see how their status as migrants, the degree of formalisation of their asylum status, and time spent in the UK factored in the way they experienced their lives and engaged in volunteering. Altogether, 30 ASRs were interviewed (16 asylum seekers and 14 refugees). Of the 30 interviewees, 20 participants were male and 10 were female. The majority were from the Middle East and North African (MENA) region with over half from Sudan, Syria, Iraq, Iran and Namibia. Twenty staff from TSOs and state agencies (Glasgow City Council, National Health Service and the Department for Work and Pensions) also contributed. TSOs included faith-based organisations, charities, and local and national non-government organisations in Glasgow. TSOs are increasingly important in providing social protection for ASRs ( Mayblin & James, 2019; Wren, 2007); thus, the perspectives of TSO employees were significant.

Semi-structured interviews have been used as a major method in researching ASRs to understand their experiences (Ferguson, 2016), because of its ability to create a healthy interaction with respondents, which helped in understanding their views and perspectives. Semi-structured interviews took up a conversational format offering richer experience and thick data which cannot be simply collected through participant observation or focus groups (Fedyuk & Zentai, 2018). Charmaz (2006, p. 25–26) emphasised that “the interviewer is there to listen, to observe with sensitivity, and to encourage the person to respond” while participants freely express their perspectives and experiences (Harvey-Jordan & Long, 2001). Interviews were typically 50–60 min in duration.^4^ Interpreters were used for interviews with individuals who did not speak English.^5^

Participant observation was also employed. This method facilitated the process of analysing ASRs’ experiences in situ because participant observation brings the researcher closer to the everyday practice of the subjects they observe (Lofland et al., 2006). Participant observation was more beneficial due to the nature of data collection than the semi-structured interviews. In which, participant observation looks further into individuals’ daily lives rather than merely accepting their self-presentations (Boccagni, 2011). Participant observation “ascertains the typicality of behaviour from on-going observations, over time and within a range of contexts, of what people do, differentiated from what they say that they do” (Boccagni & Schrooten, 2018, p. 216). Observation occurred in several TSOs such as charities, integration networks and faith-based organisations that help ASRs in Glasgow. It was conducted during drop-in sessions, community meals, food bank days, gardening activities and so on. Interactions between ASR volunteers, organisations and the local community within the organisational settings would be otherwise inaccessible through individual interviews and group discussions (Barbour & Schostak, 2005). Central to the participant observation was the first author’s personal volunteering with four TSOs that provide support to ASRs, where ASRs also performed volunteering roles. Undertaking participant observation while volunteering at organisations assisted with identifying how volunteering activities are organised and prioritised, and the nature of volunteering, type of activities involved and level of engagement.

Data was collected using audio recording and fieldnotes. Interviews were transcribed by the first author. Data was thematically analysed and coded with the use of NVivo. Participants were assigned pseudonyms for confidentiality.

This research received ethical approval from the University’s Social Sciences Departmental Research Ethics Committee and the Faculty of Arts and Sciences Research Ethics Committee. Participants gave informed consent to indicate that they understood the nature of the research, what it meant to participate in this research, and knew their rights, such as their right to decline participation and withdraw (in this case up until 28 days after the interview). Participants were given time to consider the project information sheet and ask questions before, throughout, and after the interview process. To facilitate informed consent, project information sheets and consent forms were provided in English, Arabic and Farsi.

## Findings

### Volunteering Motivation and Patterns

A primary observation was that every local organisation assisting migrants have at least two or three ASRs fulfilling a role as volunteers. The presence and distribution of TSOs and the volunteering opportunities offered throughout Glasgow have increased the likelihood of ASRs’ volunteering. Mohan and Bennett (2019) stress that although there are different levels (local, regional and national) of organisations, there is a positive correlation between the likelihood of volunteering and the distribution of charitable organisations. All those who volunteer are still receiving or have received support from those respective organisations. Being a beneficiary of those organisations reflects the existing trustworthy relationship, social connections, a sense of belonging and solidarity expressed from those who work in TSOs. Calò et al. (2021) note that TSOs provide a safe and trusted environment for use. ASRs’ preference to volunteer where they receive(d) services also reflects the importance of familiarity and comfort. Thus, a conducive environment has been created since their role as beneficiaries contributed to ASRs’ decision to volunteer with those organisations.

Three primary narratives of ASRs’ volunteering emerged in this study: helping others, helping themselves, and volunteering as not useful. Volunteering is the key active endeavour that assists many ASRs to help themselves as they utilise it as a strategy and coping mechanism to help them navigate challenges in their new society (discussed further in the following sections). ASRs did not possess adequate resources to directly support people in need and so they engaged in volunteering activities to contribute to the organisations that do have resources to help others. Further to feeling like they are doing something meaningful, helping others through volunteering created a pathway that enabled ASRs to be altruistic. Resultingly, participants additionally gained self-satisfaction through volunteering, which also positively affected their wellbeing.^6^ As illustrated in this quote:

I like it [volunteering] because I grow in helping society. I used to help my community. We help elders who are neighbours, children, so the way our values and principles are helping each other. So for me, it is normal because even I came from charity family. My mom does charities; she helps IDPs [internally displaced persons] in my country (Alimah - F, R, Sudan, 25–30).

Alimah’s comment reflects how migrants often derive from cultures, societies, and communities where altruism and helping others is embedded in their identity. Thus, when moving to new a place, these embedded altruistic characteristics travel with them. Similarly, viewing volunteering “as a good thing to do”, reflects a fundamental belief that helping others is an important facet of these individuals’ core being. While volunteering is a key strategy for ASRs to contribute to their society and feel a sense of belonging, the majority of the participants reported that altruistic aims were not the purpose for their volunteering (this is discussed in detail in the following sections).

For some participants, however, volunteering was not considered to be a meaningful activity. For example, when asked about volunteering, Adiel (M, AS, Namibia, 35–40) said:

What is the point of volunteering? Is that full-time work? Are they going to pay enough money? No. I don’t see any benefit in it. I think it is just a waste of time. Others may have a different opinion, but this is mine.

Adiel’s lack of interest in volunteering was shaped by the fact that there was no formal recognition of asylum seekers’ volunteering. Therefore, the perceived and factual differences between volunteering and formal employment factors in determining ASRs’ decision to volunteer. This demonstrates that while some asylum seekers acknowledged the everyday mundane and material benefits of volunteering, others ignored it due to its voluntary nature and a lack of perspective on its overall benefits.

ASRs have different individual motivations and experiences of volunteering, yet certain demographic characteristics do seem to have some influence. Among those who volunteer, a key observation was that the majority are asylum seekers and a smaller number are refugees. Despite participants having different reasons for volunteering, asylum seekers’ involvement could be the result of their inability to engage in formal employment due to the immigration controls; thus, their reasoning may be to volunteer to do something meaningful or contribute to their society and build a future. Asylum seekers complained about having no opportunities to engage in meaningful activities due to restrictions placed on their capacity to engage in paid work. Refugees, meanwhile, appeared less inclined to volunteer because they have the right to work. They also have to actively look for jobs and engage in other activities (i.e. language classes and placements) agreed as part of their claimant commitment attached to the Jobcentre benefits, which requires more of their time.

Furthermore, it appeared that people of similar ethnic groups tended to volunteer in certain places at certain times. There could be various reasons for this pattern of volunteering among ASRs. Firstly, since ASRs have strong informal networks, the organisations could have been recommended to them by others from within their social networks. Some participants reported having joined certain organisations because of their friends. Secondly, it could be easier to join an organisation where there are volunteers who speak the same language and understand cultural or religious beliefs. Finally, ASRs prefer to be in places where there are other people with whom they feel similar; thus, people of relatively homogenous ethnicity being the majority of the attendees at an organisation could increase the likelihood of volunteering.

The majority of ASRs observed volunteering were middle-aged. This appears to be contrary to the National Council for Voluntary Organisations’ findings that people aged 65–74 are most likely to regularly volunteer. One reason for this difference could be that the majority of those who claim asylum fall into the middle-aged category. None of the ASR volunteers—in organisations being observed—was aged over 50. Although it cannot be generalised, older ASRs might not be interested in volunteering because of their mature age, since volunteering requires travel to where they volunteer, active involvement and sometimes many hours of work.

### Gaining Familiarity and Social Connectivity

Reasons vary for ASRs’ volunteering, such as a route to employment, self-improvement, and altruistic reasons (as previously mentioned); however, a general observation throughout the fieldwork was that volunteering provided opportunities for ASRs to gain familiarity and supplement their subsistence needs. As Tan, a charity worker, explained:

We try to provide some social outcomes as well where we have volunteer days away. We visited the parliament recently. We do Christmas activities, we try somewhere to eat, we try to celebrate festivals. We try to let them become aware of social occasions within their budgets so free meals, concepts like that we would accompany them or we just let them know where they are. We are doing a drum workshop, totally social but the drum classes are going to start in October as well. Sometimes it is not really what they want to do because they are quite isolated and they have got nothing to do. So, we try to provide social information and social opportunities as well as the basic households goods and clothing that we provide.

From the perspectives of volunteers’ motivations to “understand” their world, volunteering enables ASRs to become actively involved in understanding and adapting to their new environment. As Tan’s comment reflects, ASRs could actively learn and understand their environment through working together with Glaswegians and other local community members, celebrating and partaking in local festivals, learning and practising the language with other volunteers and staff in organisations and learning the importance of a sense of community through the organisations’ activities. ASRs’ volunteering assists them to experience Glasgow as a welcoming society, where people make the effort to listen to them and see them as individuals who need to get to know their locality. Importantly, volunteering highlights that locals who support volunteering for ASRs are more accessible in terms of social connections and getting networked in the new environment.

So, in some respect, they kind of get the same welcoming as anybody would get but I think we work hard at trying to make sure that refugees who are part of our organisation are included and valued as anybody else who comes. They participate and create the [name of the organisation] for what it was. They have agency; they have opportunities for voices like anybody else. I think that is difficult sometimes, [we are] often conscious in the volunteer meeting in the morning that is much easier for some people to speak up than others if you know English. We would try various things to make that possible. So, one of the reasons why we breakdown the volunteer meeting into smaller teams is that we thought that having people talking in smaller groups might be easier to participate (Sadie - Charity Worker).

ASRs have generally lacked confidence due to their immigration status, lacked English language speaking abilities, work experience and familiarity with their new locality (Ager & Strang, 2008). Thus, it is significant that for many participants who volunteer that their volunteering contributed to them gaining confidence to engage in activities, work with others and assume responsibilities. Performing specific roles and responsibilities as a volunteer and contributing to organisational activities boosted ASRs’ self-confidence and self-esteem. For example:

There is no such thing like a boss here in our charity. We have volunteers and something called collective members. Some people have been working in our charity constantly. We do everything together and we share our tasks. Everything is discussed together, and we vote. If someone votes against something, then the thing does not proceed. So, we as volunteers decide what to do and what not to do. In that way, we feel the importance of our role and feel confident you know because they listen to me and let me contribute (Aliyah - F, AS, Sudan, 20–25).

We also found that volunteering in TSOs provided a trusted environment for ASRs to increase their knowledge about the existing support system, increase their confidence in accessing such services and adapting to their environment. Sometimes this information was received from second-hand participation in different activities in the charities where ASRs volunteer.

For example, Amina (F, R, Sudan, 40–45) stated:

I was volunteering in a charity and they offered lots of training. For example, there was training from NHS about how to talk to your children for parents who have kids in the UK. I mean people are from different cultures. So, I think it is important to know the level of importance given to children in this country.

Volunteering, in another regard, is vital to promote social connections that create opportunities for ASRs to develop intimate and reciprocal friendships. Social connection has been a critical issue around integration and inclusion when asylum seekers are dispersed to a new location without any existing social networks (Strang & Quinn, 2019). Indeed, Stewart (2005) has stressed that asylum seekers may experience vulnerability in the community due to having a lack of social connections. Participants who volunteered in charities reported having increased opportunities to meet others through involvement in integration activities, community-based events, drop-ins and other activities, which were attended by both ASRs and local community members. Takudzwa (M, AS, Zimbabwe, 40–45) stated:

I now have a lot of contacts with people, you know, from all the voluntary work that I do, like the choir and just going places. I also volunteer at an organisation so when we do events, I meet a lot of people and we also go out to the community; we help the community.

Participants’ narratives about making social connections reflected the importance of social bonds and social bridges. In one regard, volunteering fostered participants’ engagement with individuals and families from co-ethnic, co-national, co-religious or other forms of similar backgrounds, which Ager and Strang (2008) have called social bonds. For instance, when Aleea (F, AS, Iraq, 35–40) arrived in Glasgow in 2017, she did not have any social networks and failed to establish any connections due to a lack of opportunities to meet others in her asylum accommodation or outside the home. However, volunteering for a charity in Govan, Glasgow, fostered interactions with others and increased her chances of forming social bonds with fellow ASRs. Aleea stated:

When I enter the charity, I start seeing people. Sometimes we don’t know [the same] languages but I try. When I tried to help, they are happy. So, in the end, we become friends. If they want anything, they come again and look for me. A woman I met asked me to come to her house to celebrate her daughter’s birthday. See, this is what happens if you volunteer. You meet people, you build friendships and look after each other.

Furthermore, participants established social bridges, networks or connections with local community members. Volunteering increased one’s chances of meeting members of their local community because locals’ involvement in charities, faith-based organisations and NGOs provided a platform for ASRs to meet and interact with locals. Volunteering promoted interactions between participants and local community members in two ways. First, in the majority of the organisations, local community members volunteered alongside ASRs. In this case, ASR volunteers worked with local volunteers to support and implement the day-to-day organisational activities such as coordinating and conducting food banks, drop-in sessions and community meals. These opportunities for ASR volunteers to interact with local community members by working as volunteers alongside them enabled the fostering of relationships. Opportunities to volunteer alongside locals also increased their feelings of community acceptance:

I don’t know about others but for me, I became friends with Glaswegians who volunteer in the same organisation too. You do things together, you talk, you share and eat together. Now, yeah, there are like three to five people that families that we visit each other here in Glasgow. We are very satisfied and comfortable with this relationship with Scottish people (Mustafa - M, AS, Iraq, 40–45).

Second, some local community members attended charity events.^7^ Regardless of their position as local volunteers or participants, local members’ presence in organisations facilitated ASR volunteers’ interactions with others. Several participants mentioned that members of these two groups exchanged opinions, information and shared their experiences over tea or coffee. This inter-group interaction particularly helped ASRs to understand locals’ opinions of migrants and vice versa.

There is a relationship barrier between people here [migrants and Glaswegians] but I can say I know how to deal with it. You go and volunteer in organisations. When you come, you do many activities, you can just see people. It is very nice and you feel very comfortable when locals come here [to the charity]. They help a lot and get involved (Amina - F, R, Sudan, 40–45).

Many participants expressed feeling afraid of getting involved in the community because of uncertainties (asylum status and temporary housing) and the associated problems of being an asylum seeker or a refugee, such as racism, prejudice and negative stereotypes. Consequently, they lacked confidence and were reluctant to engage in various local community activities other than volunteering (e.g. sports, cultural and social activities), which appears here as a safe space for mixing and creation of initial social ties. However, participants’ active engagement when it occurred assisted them to boost their self-confidence and self-esteem and to become involved and engaged in their new locality. ASRs’ fear of participation is reflected in the following quote:

[ASRs] are not confident enough. Like I was [not confident] in the past. I didn’t talk much with people or attend any events. But after some time, I started to volunteer. After that, I tried to talk to many people, go to different organisations, participate in activities. I learnt from working with organisations and groups. So, now, I always say to them [other ASRs]: don’t worry, if someone is talking to you, talk to them and they cannot punish you for trying to talk. Some people are not confident enough. It’s not a cultural or religious reason but a personal thing. That is why volunteering is a good start. You work with a few people, make yourself comfortable, learn from them; you can learn English and then you feel good to go out and talk with others (Amina - F, R, Sudan, 40–45).

Social connections or social networks are a primary benefit of volunteering. Engagement in formal and informal activities through TSOs indicates ASRs’ active participation in their community, while the social networks formed reflect an individual’s access to social capital. Indeed, as Vickers (2016) emphasised, building social capital has connected refugees as volunteers and users with TSOs and community. In other words, social capital derived through their volunteering reflects, to a degree, ASRs’ opportunities and ability to count on others for social and emotional support in times of need. Thus, volunteers could obtain first-hand information from trusted organisations and professionals rather than obtain information through word-of-mouth stories, or second-hand information while they performed different tasks in these charities.

### Suplementing Subsistence Needs: the Significance of Material and Financial Gains for Inclusion

As mentioned, volunteering is a tool for ASRs to supplement their subsistence needs. ASRs’ engagement with volunteering within TSOs as a survival strategy largely reflects the hardships and limitations that they face due to the inadequate financial support from the social support system. Their subsistence is characterised by significantly limited financial and material resources, which also restrict their urban mobility and overall connectivity. Asylum seekers are considered destitute as individuals without sufficient resources or the financial capacity to meet basic subsistence needs (Randall, 2015); thus, the Home Office provided (at the time of the data collection) financial support of £37.75 per week for asylum seekers to purchase food, clothing and toiletries. The amount of asylum allowance offered, however, was revealed to be a cause of frustration and humiliation among participants who said that despite this allowance, they struggled to meet their basic needs.

For example, Takudzwa (M, AS, Zimbabwe, 40–45) commented on the amount of asylum allowance, saying, “it is like insulting people because if you give me £35 now, it will be spent within five hours. Then how do I survive?”. Crawley et al. (2011) suggested that state-enforced destitution is a key UK immigration policy outcome to disincentivise asylum seekers from seeking refuge in the UK. However, this appears to only affect the experience of those asylum seekers already in the UK. While none of the participants in this study were disincentivised, they often expressed disappointment with the asylum allowance. For example:

Can you manage your life with this £37.75 per week? (Babar - M, AS, Pakistan, 30–35).

What do you think? Does that [sound like] enough [money] for people to live here? (Aleea - F, AS, Iraq, 35–40).

Asylum seekers expressed a willingness to engage in formal employment to earn their own income and avoid being dependent on the state; however, the current system restricts them from seeking formal employment. Their willingness but the inability to work and earn income also caused frustration, as Aleea (F, AS, Iraq, 35–40) explained:

They [the UK government] don’t provide for us, but why don’t they let us work and then we can pay for ourselves? Okay, you can’t pay for me so let me work and I will pay for myself. You don’t let me work and you make me lazy. You give me £35 for a week or £5 a day, but the daytime bus ticket itself is £4.50. So, how do we survive?

As a result of asylum seekers struggling to survive due to inadequate financial support and formal employment restrictions, they utilise opportunities and benefits afforded by volunteering in TSOs to supplement their resources and meet their subsistence needs. Although ASRs may volunteer in TSOs to assist those organisations to deliver services and contribute to community support, our findings suggest volunteering is also a tool enabling ASRs to secure material (goods) and modest financial support (e.g. reimbursement of travel expenses).

The Home Office (2019) guidelines on permission to work and volunteering for asylum seekers states that those who volunteer might be reimbursed for expenses that occurred during their volunteering (i.e. travel and food expenses). Therefore, volunteers in TSOs are often compensated for travel costs related to their volunteering activities. According to participants, the money received associated with volunteering, which is approximately £5 per day, was paid to subsidise volunteers’ travel expenses to and from the place where they volunteer. Some participants who volunteer indicated that this financial remuneration was their primary motivation for volunteering, while others highlighted the other benefits of volunteering (discussed in the other sections):

To be honest I don’t want to volunteer; I want to do proper work, but my situation is difficult because of my asylum status. No work and not enough money. So, charities here give you some money for volunteering. It is not much but, you know, it helps me to cover some costs that I couldn’t manage with my asylum allowance (Abeo - M, AS, Nigeria, 40–45).

Although the purpose of financial support given to volunteers is to cover volunteers’ transport costs, asylum seekers use this money in a variety of ways to meet or supplement their subsistence needs and improve their interconnectivity and quality of life. Those who saved some money from their volunteering travel reimbursement could spend it on food, household items and utilities. For example:

I save money from my volunteering and spend it on food. Some foods are expensive here. I can’t buy them with my asylum money. So, I save for some weeks and buy some good food once a month. Something healthy and nice (Aliyah - F, AS, Sudan, 20–25).

I sometimes use my volunteering money to top-up my phone. Sometimes I save for a couple of weeks and buy an expensive top-up [data package] so I can use internet without any problems (Babar - M, AS, Pakistan, 30–35).

Several participants used travel money from volunteering to purchase bus tickets for travel unrelated to the volunteering:

Sometimes we have to take public transport to go to the doctor or Serco [asylum housing provider] or the Home Office but it is not easy to use our asylum money to buy bus tickets. A daytime ticket will take away £5, which we need to [otherwise] spend on food. So, I use the volunteering money from my charity to cover bus tickets (Dalilah - F, AS, Egypt, 30–35).

Participants’ narratives indicate that the asylum allowance does not cover any contingencies, even minimal, such as travelling to the Home Office for their asylum application. One participant explained that receiving £5 per day for 5 days per week volunteering meant he received £25 from the volunteering travel expenses reimbursement. Since the volunteering travel reimbursement is paid daily, and weekly bus passes purchased in advance are cheaper (£16), he bought the weekly bus pass using his asylum allowance and then reimbursed himself to pay for living costs from his volunteer financial support. Purchasing a weekly bus pass in advance was beneficial since it meant that overall, each week, he saved money. An additional benefit was that a weekly bus pass enabled him to travel more frequently throughout that week for various reasons without further travel costs.

The previous examples illustrate how the financial support (travel expenses reimbursement) given to volunteers motivates some participants to volunteer and there was additional evidence to suggest that for some ASRs volunteering offers access to material benefits such as food, clothes and household items. In general, participants came to the UK without many material possessions.^8^ Considering a lack of access to material support (for example food and clothes, due to a limited asylum allowance) in their new environs, supplementing material and financial resources through volunteering contributes to them feeling as if they are successfully resettling. As Noys (2016) stated, through social practice, a sense of the living and feeling has been transferred onto objects, which in turn emphasises the value attached to the material object. Indeed, many charities play a crucial gap-filling role by providing material support to ASRs in need, e.g. food banks, clothes banks and household items, reflected in the following quotes:

My wife works in a charity as a volunteer, so she gets things from there. When we need something, we just go and get it (Mustafa - M, AS, Iraq, 40–45).

I have to say that we [volunteers] have an advantage through volunteering; that is, our easy access to things in our charity. Because you are a volunteer, you can ask for more stuff, like extra packs of pasta or rice. Oh! We can also get leftover fresh fruits and vegetables after food banks (Namazzi - F, AS, Uganda, 30–35).

Asylum seekers frequently utilise food banks and dry rations from charities, although the quantities of free products an individual can receive at a particular time is limited. However, findings suggest that asylum seekers who volunteer collect more items than non-volunteering asylum seekers who use the food banks. Participants who volunteered had access to food and other items both during and after food bank days and outside of the organisations’ business hours and this increased their access to provisions. Therefore, volunteers are more conveniently positioned to access food and other provisions rather than having to wait—as non-volunteering asylum seekers do—for specific food bank days.

While asylum seekers largely engage in volunteering as a strategy to overcome the impacts of inadequate state provision, refugees also gain material benefits through volunteering. Participant observation revealed that volunteers in a charity had the opportunity to go through and collect suitable items before and after the specific drop-in hours. On Wednesdays, a charity had drop-ins from 10 am to 2 pm for items such as clothing and small household items. On three occasions, Alimah (F, R, Sudan, 25–30), a refugee and volunteer, was observed collecting different items.^9^ She took them and put them aside before the drop-in commenced. One day she explained the advantages of working as a volunteer in a charity while pointing at some of the household items she had collected before the drop-in opened:

These are very good. I want them. They [individuals who attend the drop-ins] will take [these things] it if I don’t take it and hide it now. That is why I come early, too.

Importantly, organisations adopt different policies and so not all the volunteers can access items outside normal food bank hours; at the same time, the volunteering domain is partly informal where ASRs can exercise some agency.

Wilson (2012) indicates that individuals’ motivation to volunteer may depend on the consumable resources and rewards, and while the author does not focus on ASRs, this comment suggests that consumable resources and rewards could motivate individuals belonging to marginalised groups to volunteer. In this research participants’ inclination to engage in volunteering for material benefits is linked to the current immigration controls and a lack of support they receive from the state. Regarding immigration controls, asylum seekers in the UK are ineligible to engage in formal employment and they have no recourse to access public funds (Mulvey, 2010; Guentner et al., 2016). Although monetary reimbursement for volunteering is modest, it has nevertheless increased asylum seekers’ (volunteers) purchasing capacity and partially contributed to the supplementation of their subsistence needs, as well as symbolically consisting of a gain when facing severe restrictions and the arduous process of settlement.

## Conclusion

We have argued that ASRs engage in activities as volunteers in many TSOs in Glasgow and that for them, volunteering is highly beneficial for their inclusion. Our findings corroborate existing literature identifying the positive benefits of volunteering in general and for ASRs more specifically. Although volunteering appears to be strongly associated with employment or labour market prospects in the migration literature, our findings highlight the significance of direct and indirect benefits of volunteering and consider the long-term impacts of volunteering in other areas of ASRs’ lives. Differently from what the existing research has identified, the benefits of volunteering in this research are not seen from a purely structural point-of-view, but from the point-of-view of ASRs and their daily, mundane experiences. Indeed, these understated experiences appear to be the flesh in the otherwise abstract framework of inclusion.

Findings further showed that ASRs are somewhat active agents when engaged in volunteering to improve their living conditions and create a future. This includes building social connections, becoming actively involved in increasing their employment prospects, altruistic views and so on. Nevertheless, the reasons for volunteering cannot be generalised and are specific to individuals. Individuals have volunteered to access resources for their survival, for self-improvement, employment prospects, building social connections, creating a sense of belonging, and or for altruistic reasons.

Examining the everyday practices of volunteering, with its supposed mundane and banal qualities, provides a unique perspective to explore a more individual outlook. The findings show strong, albeit understated links to the new environment. This familiarity is particularly evident in terms of establishing initial connections and learning about mundane as well as functional aspects of daily life and work. We have found that volunteering has been a key opportunity for many ASRs to develop social capital by establishing connections through their volunteering. As evident from our research, the social capital formation has shaped ASRs’ individual actions, their ability to negotiate access to social and material interests and expected membership in their local community (Vickers, 2016). The secondary material and financial benefits from volunteering are intrinsically linked to the processes of adaptation and inclusion as they increase the ASRs’ daily urban mobilities, their digital interconnectivity, while the material items they acquire enhance their homes and symbolise small victories in the face of harsh restrictions.

Investigating how and why ASRs engage in volunteering—as we have done here—shows that ASRs essentially problematise the definition of volunteering provided at the outset of this paper. Rather than work for no monetary or material gain, ASRs volunteer for TSOs because these organisations fill the gap left by the state in providing for ASRs’ essential survival, inclusion and integration needs, whether they be social, material and economic. It is also ambiguous as to whether ASRs—as vulnerablised individuals—engage with volunteering in a way that is implied by the term “free will”. Asylum seekers are not allowed to work, refugees struggle to find work, and the definition of volunteering is based on a non-profit idea of engagement in productive activities. However, ASRs benefit indirectly from volunteering by generating financial and material resources due to formal and informal access to volunteering-related resources. These benefits are an important part of ASRs’ settlement in the new environment, their wellbeing, the improvement of their circumstances, and engagement with the every day and expression of their agency in the process of integration.

While volunteering motivations to supplement their subsistence needs through available material and financial benefits could be opportunistic in appearance, we suggest that this has to be understood parallel to the policy-imposed liminalities against asylum seekers in the UK. Throughout this research, ASRs exercised agency through self-governing their whole volunteering by decision-making throughout the process rather than partaking in activities that are being imposed upon them; their approach to the above-mentioned benefits should be seen within this framework.

Furthermore, this analysis emphasises the role TSOs play in providing volunteering opportunities and supporting the call for state investment (financial support) into local organisations to strengthen volunteering prospects. As the aim of the Scottish integration policy is to promote and facilitate ASRs’ inclusion and participation, acknowledging the direct and hidden benefits, state support would enable the TSOs to provide more opportunities. Volunteering opportunities is a significant by-product of TSOs that are assisting ASRs in their localities.

A future direction from this project will be to understand the impact of COVID-19 pandemic on opportunities for ASRs to continue the same level of volunteering involvement and to examine the post-pandemic challenges ASRs have faced.

## Footnotes

1 At the time of the research, asylum allowance was set at a rate of 70% of mainstream welfare benefits, which was around £39 per week for single adults.

2 https://bills.parliament.uk/bills/3023.

3 The authors plan future publication on this topic.

4 Interviews were conducted mainly in libraries.

5 For example, those who spoke in Arabic and Farsi languages.

6 Of course, it could be argued that gaining self-satisfaction and improving one’s wellbeing as “rewards” for volunteering and thus counters claim of altruism.

7 For example, to access resources or to pass time, as attendees and as helpers.

8 Except for their bag(s) of clothes, which were unsuitable for cold weather.

9 Such as clothes, drying rack, wall mirror, cutleries and plates.
